# Short-term outcomes of transanal completion total mesorectal excision (cTaTME) for rectal cancer: a case-matched analysis

**DOI:** 10.1007/s00464-018-6280-3

**Published:** 2018-07-02

**Authors:** T. W. A. Koedam, M. Veltcamp Helbach, M. Penna, A. Wijsmuller, P. Doornebosch, H. L. van Westreenen, R. Hompes, H. J. Bonjer, C. Sietses, E. de Graaf, J. B. Tuynman

**Affiliations:** 1 Department of Surgery, VU University Medical Center, Boelelaan 1117, 1081 HV Amsterdam, The Netherlands; 2 Department of Surgery, Hospital Gelderse Vallei, Ede, The Netherlands; 3 Department of Colorectal Surgery, Churchill Hospital, Oxford, UK; 4 Department of Surgery, IJsselland Hospital, Capelle a/d IJssel, The Netherlands; 5 Department of Surgery, ISALA Hospital, Zwolle, The Netherlands; 6 Postbus 7075, 1007 MB Amsterdam, The Netherlands

**Keywords:** TAMIS, TaTME, Completion, Rectal surgery, Outcomes

## Abstract

**Background:**

Local excision of early rectal tumors as a rectal preserving treatment is gaining popularity, especially since bowel cancer screening programs result in a shift towards the diagnosis of early stage rectal cancers. However, unfavorable histological features predicting high risk for recurrence within the “big biopsy” may mandate completion total mesorectal excision (cTME). Completion surgery is associated with higher morbidity, poorer specimen quality, and less favorable oncological outcomes compared to primary TME. Transanal approach potentially improves outcome of completion surgery for rectal cancer. The aim of this study was to compare radical completion surgery after local excision for rectal cancer by the transanal approach (cTaTME) with conventional abdominal approach (cTME).

**Methods:**

All consecutive patients who underwent cTaTME for rectal cancer between 2012 and 2017 were case-matched with cTME patients, according to gender, tumor height, preoperative radiotherapy, and tumor stage. Surgical, pathological, and short-term postoperative outcomes were evaluated.

**Results:**

In total, 25 patients underwent completion TaTME and were matched with 25 patients after cTME. Median time from local excision to completion surgery was 9 weeks in both groups. In the cTaTME and cTME groups, perforation of the rectum occurred in 4 and 28% of patients, respectively (*p* = 0.049), leading to poor specimen quality in these patients. Number of harvested lymph nodes was higher after cTaTME (median 15; range 7–47) than after cTME (median 10; range 0–17). No significant difference was found in end colostomy rate between the two groups. Major 30-day morbidity (Clavien–Dindo≥ III) was 20 and 32%, respectively (*p* = 0.321). Hospital stay was significantly longer after cTME.

**Conclusion:**

TaTME after full-thickness excision is a promising technique with a significantly lower risk of perforation of the rectum and better specimen quality compared to conventional completion TME.

Early rectal cancer is increasingly treated with transanal minimal invasive surgery (TAMIS). With the implementation of population screening programs for colorectal cancer and longer life expectancy, the incidence of elderly patients being diagnosed with early rectal cancer increases. Rectal preserving therapy is appealing for these patients as radical rectal surgery has been correlated with higher colostomy rate, higher morbidity, and poorer functional outcomes [1–8]. Commonly used platforms for full-thickness local excision include transanal endoscopic microsurgery (TEM), transanal endoscopic operations (TEO) and the GelPoint® Path port which offer a safe and curative therapy when performed for large rectal adenomas and low-risk T1 carcinoma, including tumors smaller than 3 centimeters in size, well-to-moderately differentiated, and without lymphovascular or submucosal Kikuchi level III invasion [9, 10].

Despite all the diagnostic modalities available, final pathology after local excision often reveals high-risk T1 or more invasive rectal cancer in 40% of the patients [11]. In those cases, completion radical surgery (cTME) is advised in most guidelines, since the risk for local recurrence in these patients is significantly increased after local excision only [12–14]. This strategy is, however, feared by many surgeons due to the potential adverse outcomes, as completion surgery is reported to be more difficult due to weakened integrity of the rectal wall due to inflammatory change or fibrosis at the previous excision site. This could lead to a poorer TME specimen quality, and increased risk of rectal perforation and local recurrence compared to primary total mesorectal excision (TME) [1–3]. Considering the difficulties of an abdominal approach in cTME, the transanal approach could be of particular benefit in these patients. Aside from better visualization, the “rectal scar” can be approached from both sides allowing more careful dissection of the mesorectum without undue traction that can lead to specimen fragmentation and/or perforation of the rectal tube. The aim of this study was to evaluate the short-term clinical and pathological outcomes of patients needing completion TaTME (cTaTME) for rectal cancer compared to cTME.

## Materials and methods

### Patients

A consecutive multicenter cohort of patients who underwent completion surgery after endoscopic full-thickness local excision, further mentioned as TAMIS, for rectal cancer was collected. A case-matched analysis was conducted to compare the short-term clinical and pathological outcome of completion transanal TME (cTaTME) with conventional completion TME (cTME), both following previous full-thickness local excision. In order to minimize potential confounding effects of baseline characteristics on pathological and short-term outcomes, case-matching was performed using the covariates gender, neoadjuvant radiotherapy, tumor height, and pathological tumor stage. The cTaTME group included consecutive patients treated for rectal cancer between January 2012 and January 2017 and matched with a historic group of patients operated between January 2000 and January 2012. Both groups were operated in one of the four participating hospitals, which are tertiary referral and teaching centers for laparoscopic and transanal TME (VU University Medical Center in Amsterdam, Hospital Gelderse Vallei in Ede, IJsselland Hospital in Capelle aan den IJssel, ISALA in Zwolle, and the Churchill Hospital in Oxford). Patients who received TaTME for a recurrence after earlier endoscopic full-thickness local excision (salvage surgery) were not included in this study. This study was approved by the institutional review board of the VU University Medical Center, Amsterdam, The Netherlands.

### Preoperative assessment

The preoperative assessment was performed according to Dutch and UK guidelines [14, 15]. The work-up before the local excision procedure included clinical evaluation, colonoscopy, biopsy, and endorectal ultrasound (ERUS) when a benign lesion was expected. When patients had a biopsy proven carcinoma, a computed tomography (CT), magnetic resonance imaging (MRI), or contrast-enhanced MRI of the pelvis was added prior to the TAMIS. When a benign lesion was expected before TAMIS, but histology revealed a carcinoma, a CT, MRI, or contrast-enhanced MRI was performed after the TAMIS. In all patients, imaging of the liver and lungs was performed to exclude distant metastasis.

### Surgical technique

TAMIS was routinely performed under general anesthesia with antibiotic prophylaxis. After positioning the patient and sterilizing the operative area, the anal platform and insufflator were applied to create pneumorectum. With the diathermy hook, a 1-cm margin of macroscopically normal mucosa was created; hereafter full-thickness transmural excision was performed.

TaTME was performed following the standardized approach described by Lacy et al. [16], under general anesthesia with antibiotic prophylaxis. The abdominal phase started laparoscopically, mobilizing the splenic flexure and proximal part of the (meso)rectum. The transanal approach is performed followed by inserting the Lonestar retractor and Gelpoint platform to close the rectum with a purse string and cleaning the closed rectum with betadine. In cases requiring an intersphincteric abdominoperineal resection, the anal platform was positioned after the intersphincteric dissection was finalized. The location of the previous TAMIS-site was always dissected under direct visualization. Finally, the specimen was retrieved through a Pfannenstiel incision or transanally when the specimen was small enough.

### Definitions

Completion surgery was defined as pre-emptive radical surgery based on pathological risk factors and without signs of local recurrence. Transanal TME was defined as dissection of the mesorectum including the previous TAMIS-site via a transanal platform with insufflation. Quality of specimen was assessed by the pathologist according to the previously published classification provided by Nagtegaal et al. [17]. Perforation of the rectum was defined as an explicit statement by the surgeon or pathologist. Experiencing difficulty during surgery was evaluated by the surgical report. Postoperative complications were graded using the Clavien–Dindo classification, in order to separate minor (Grade I–II) from major complications (III–V). An anastomotic leakage was defined as associated clinical symptoms and raised inflammatory markers (e.g., leukocytosis, fever, pain) with contrast extravasation on CT-scan and/or a defect visible during endoscopy. A presacral abscess was defined as associated clinical symptoms with a pelvic collection visible on radiological evaluation.

### Data

Data extraction from patient charts was performed prospectively for the cTaTME group and cTME group by local physicians and recorded anonymously, including baseline characteristics, preoperative diagnostics, operation details, and pathology following local excision, operation details and pathology after completion surgery, and postoperative course. Case–control analysis between cTaTME and cTME was performed by matching for gender, tumor height, neoadjuvant radiotherapy, and tumor stage. Dichotomous and categorical values were analyzed using the Pearson Chi^2^ test. For normally distributed continuous data, mean values with range are reported and analyzed using the independent student *t* test. For non-normally distributed data, median values with range are reported and analyzed using the Mann–Whitney *U* test. Case-matching and all other analyses were performed using IBM SPSS statistics, version 23.0.0.0 (IBM corp., NY, USA).

## Results

### Patient and tumor characteristics

A prospective cohort of completion surgery was obtained from 88 patients in 4 centers. A total of 25 patients were operated by cTaTME for rectal cancer (out of 312 patients operated by TaTME in this period) and matched to 25 out of 63 patients after cTME. The patients and tumor characteristics are summarized in Table 1. Full-thickness local excision was performed in all patients. Postoperative complications after the TAMIS operation were observed in two patients in the cTaTME group (one patient with fever due to unknown cause was given antibiotics and one mild stenosis not requiring dilatation), and five patients in the cTME group (two patients with fever due to unknown cause requiring antibiotics and three with bleeding from the operation site, not requiring re-intervention). Histology after TAMIS in the cTaTME and cTME groups showed a high-risk T1 in 11 (44%) and 7 (28%) patients, respectively, and a more advanced lesion in 14 (56%) and 18 (72%) patients, respectively (Table 1). Based on the histology, all patients needed completion surgery for curative management according to the current Dutch and UK Rectal Cancer Guidelines.

**Table 1:** Baseline and initial tumor characteristics

	cTaTME	cTME	*p* value
Patients, *n*	25	25	
Male, *n* (%)	13 (52)	16 (64)	0.567
Age (years)^a^	69 (55–82)	66 (47–84)	0.385
Tumor distance from AV on MRI			0.148
High (≥ 10 cm)	5 (20)	9 (36)	
Mid (5 cm ≤ and < 10 cm)	13 (52)	14 (56)	
Low (< 5 cm)	7 (28)	2 (8)	
Anterior location of rectum, *n* (%)	7 (39)	8 (33)	0.382
Tumor size (mm)^a^	2.5 (0.3–8.0)	2.5 (1.0–8.0)	0.603
Pathological tumor stage after TAMIS, *n* (%)			0.156
T1	12 (48)	7 (28)	
SM1-2	2	1	
SM3	7	4	
Unclear	3	2	
T2	11 (44)	11 (44)	
T3	2 (8)	7 (28)	
Lymphovascular invasion, *n* (%)	7 (28)	5 (20)	0.742
Radical local excision, *n* (%)	6 (25)	12 (48)	0.140
Neoadjuvant radiotherapy, *n* (%)	7 (28)	8 (32)	1.000

*AV* anal verge, *TAMIS* transanal minimal invasive surgery

^a^Values are median number with ranges in parentheses

### Completion surgery

In both groups, 30% of the patients received radiotherapy before completion surgery. In 19 patients (76%) of the cTME group and 16 patients (64%) of cTaTME group, a low anterior resection with primary anastomosis and diverting ileostomy was performed in 13 (81%) and 16 (84%) patients, respectively. Distribution of other resections is shown in Table 2. In the cTME group, open approach was used in 9 patients (36%) and the other patients were operated by laparoscopy. Perforation of the rectum at or near the previous TAMIS-site occurred in 7 patients (28%) and difficulties experienced due to fibrosis and/or inflammation at the previous TAMIS-site were reported in 17 patients (68%). Conversion occurred in three patients (19%); two patients were converted to complete dissection at the previous TAMIS-site through a Pfannenstiel incision and one patient was converted from a laparoscopic low anterior resection to Hartmann’s colostomy as construction of the anastomosis failed due to insufficient quality of the intestinal wall. In the cTaTME group, laparoscopic approach to the abdominal phase was used in all patients. In two patients (8%), conversion to laparotomy was decided due to pulmonary problems. In one patient (4%), a perforation of the rectum occurred at the previous TAMIS-site. In 32% of patients, difficulties were encountered at the previous TAMIS-site. Median interval to completion surgery was 9.4 weeks (range 3.1–38.7) in the cTME group and 9.4 weeks (range 3.4–26.6) in the cTaTME group. Timing of the completion varied due to healing process of the TAMIS-site evaluated endoscopically, need for neoadjuvant radiotherapy, occurrence of complications after TEM, and the type of operation.

**Table 2:** Completion surgery details

	cTaTME	cTME	*p* value
Interval TAMIS to TaTME (weeks)^a^	9 (3–27)	9 (3–39)	0.303
Procedures, *n* (%)			
Low anterior resection	16 (64)	19 (76)	0.484
Deviating ileostomy	13	16	
Hartmann’s procedure	5 (20)	4 (16)	
Abdominoperineal excision	4 (16)	2 (8)	
Operation time (min)^a^	239 (120–375)	185 (97–360)	0.081
Blood loss (mL)^a^	100 (50–1500)	350 (200–1800)	0.258
Conversion to laparotomy (n/N)	3/16	3/25	0.362
Perforation of the rectum	1 (4)	7 (28)	0.049
Difficult case reported by surgeon	8 (32)	17 (68)	0.023

*TaTME* transanal total mesorectal excision, *TAMIS* transanal minimal invasive surgery

^a^Values are median number with ranges in parentheses

### Pathology

Comparing the cTME and cTaTME groups, all specimens had free resection margins with good quality of the mesorectum in, respectively, 40 and 88%, intermediate quality in 32 and 8%, and poor quality in 28 and 4% of the patients, *p* = 0.001. Median lymph nodes harvest were 10 (0–17) and 15 (7–47), respectively, *p* < 0.001. Final tumor stage was comparable between both groups, including a similar proportion of patients with positive lymph nodes (Table 3). No significant differences were found in quality of specimen or perforations between patients receiving completion surgery within 9 weeks after local excision compared to patients who were operated 9 weeks or more after local excision.

**Table 3:** Pathology after completion surgery

	cTaTME	cTME	*p* value
Differentiation, *n* (%)			0.667
Well to moderately	23	21	
Poorly	2	4	
Quality of the mesorectum, *n* (%)			0.001
Good	22 (88)	10 (40)	
Intermediate	2 (8)	8 (32)	
Poor	1 (4)	7 (28)	
Lymph nodes^a^	15 (7–47)	10 (0–17)	< 0.001
Radical resection margin, *n* (%)	25 (100)	25 (100)	1.000
Final staging according to AJCC, *n* (%)			0.848
I	13 (52)	12 (48)	
II	2 (8)	4 (16)	
III	10 (40)	9 (36)	

*TaTME* transanal total mesorectal excision, *AJCC* American Joint Committee on Cancer

^a^Values are median number with ranges in parentheses

### Postoperative course

Overall morbidity was 68% after cTME and 40% after cTaTME, with major morbidity (Clavien–Dindo ≥ III) 32 and 20% (*p* = 0.321), respectively (Table 4). Major morbidity in the cTME group included three patients with an anastomotic leakage requiring a Hartmann’s procedure, one patient with a myocardial infarct requiring percutaneous recanalization of the coronary artery, one patient with bleeding requiring re-operation, two patients with an evisceration, and one patient had a retracting ileostomy requiring revision. In the cTaTME group, one patient required laparoscopic washout and pelvic drainage for an anastomotic leak 2 days after low anterior resection with deviating ileostomy, three patients developed a presacral abscess after a Hartmann requiring drainage, and one patient with a presacral abscess also had superficial necrosis of colostomy requiring revision. No patients died during the postoperative period in this study. Median hospital stay after cTME surgery was 15 days (4–43) and 8 days (range 3–45) after cTaTME, *p* = 0.004. No significant difference was found in postoperative complications between patients receiving completion surgery within 9 weeks (*n* = 29) after local excision compared to patients who were operated 9 weeks or more after local excision (*n* = 21).

**Table 4:** Postoperative course

	cTaTME	cTME	*p* value
Postoperative morbidity/mortality, *n* (%)	10 (40)	17 (68)	0.088
Minor (CD I–II), *n* (%)	7 (28)	9 (36)	0.538
Stoma-related problems	2	2	
Urinary problems	3	1	
Pulmonary problems	0	1	
Surgical Site Infection	0	1	
Presacral abscess	1	2	
Fever with unknown cause	1	2	
Major (CD III–IV), *n* (%)	5 (20)	8 (32)	0.321
Anastomotic leakage	1	3	
Presacral abscess	3	0	
Platzbauch	0	2	
Bleeding	0	1	
Stoma-related problems	1	1	
Cardiac problems	0	1	
Mortality (CD V)	0	0	
Hospital stay (days)^a^	8 (3–45)	15 (4–43)	0.004
Rate of readmission	4 (16)	5 (20)	1.000
Rate of re-operation	4 (16)	6 (24)	0.725

*CD* Clavien–Dindo classification

^a^Values are median number with ranges in parentheses

## Discussion

In more difficult pelvic surgery cases, including redo-operations and patients with adhesions due to endometriosis, TaTME has already shown promising results [18, 19]. Since completion TME is associated with higher morbidity, poorer specimen quality, and less favorable oncological outcome compared to primary TME, the transanal approach could improve the accuracy of the TME dissection. This case-matched, prospective, multicenter cohort study shows that following full-thickness local excision for rectal cancer, completion transanal TME has been proven to be superior to conventional completion TME in terms of quality of specimen, amount of rectal perforations, harvested lymph nodes, and hospital stay.

Full-thickness local excision is an accepted therapy in order to save the rectum in early cancer management. Unfortunately, preoperative staging using ERUS or MRI remains unreliable for early rectal cancers to distinguish T1 from T2 cancer as well as to diagnose other risk factors for recurrence (e.g., depth of submucosal invasion, lymphatic or vascular invasion, tumor budding) preoperatively. As a result, a subgroup of patients treated with local excision will need additional surgery based on the precise pathological assessment of the TAMIS specimen. The local excision is therefore sometimes regarded as a staging “big biopsy” instead of a final treatment. However, the increased morbidity (53%) and risk of local recurrence (HR 6.8; 95% CI 2.7–17.0; *p* < 0.0001) associated with completion radical surgery compared to immediate TME is of concern [1–3]. Completion TME is also associated with increased perforation of the rectal wall (up to 20% of patients), prolonged operating time, and increased rate of permanent stoma rate compared with primary TME [1–4]. Difficulty of completion surgery is mainly suggested due to fibrosis or inflammation of the perirectal tissue at the previous TAMIS-site in our analysis. When comparing quality of the mesorectum after laparoscopic or open completion TME with our results, better pathological outcome is observed, showing poor quality of the mesorectum in 28% of the patients after cTME versus only 4% in this study after cTaTME. Poor quality of the mesorectum and (slightly less) intermediate quality mesorectum are associated with an increased risk of local recurrence, and therefore an important parameter to consider when comparing oncological resection of rectal cancer [20]. Iatrogenic perforation of the rectal tube also correlates with local recurrence [21]. Decreasing this risk from 20% in an earlier study and 28% in our cTME group to 4% in our cTaTME group seems very promising [2]. These outcomes are comparable to previous published results of primary radical rectal surgery [22–24].

Completion TME is also associated with higher colostomy rate compared to primary TME (51 vs. 46%, OR 2.5; 95% CI 1.30–4.86) [1, 3, 4]. In TME surgery “from above” surgeons may be restricted in constructing an anastomosis in the previously operated rectum, and an increased distal margin is necessary to include the TAMIS-scar, which leads to an increased rate of permanent colostomies [4]. Especially when full-thickness excisions occurred in the distal rectum, abdominoperineal excisions are performed to avoid breaching the specimen as the scar is strongly adherent to the pelvic floor [4]. This is impossible to avoid with the abdominal approach, but feasible now with TaTME as a combined approach, thanks to the placement of a purse string in cTaTME just below the TAMIS-scar, dissecting under direct vision and a circular stapled coloanal anastomosis being safely made transanally. In this matched cohort analysis, the colostomy rate did not differ significantly between the two groups and may be due to the surgeons being at the early stages of their learning curve for cTaTME in this current cohort. Furthermore, in some patients preoperative function was assessed as inadequate for restorative surgery and therefore received a definite colostomy despite tumor height. Longer follow-up and larger sample size is needed to evaluate differences of sphincter-saving procedure in more detail.

Despite the correction for potential selection bias, the small sample size should be noted as a limitation of this study. Identification of predictive factors or multivariate analysis for worse pathological outcome is therefore not possible. Due to substantial differences in follow-up in cTME versus cTaTME, the focus of this article is only on short-term results. Long-term follow-up, including oncological and functional outcomes, will be evaluated after 36 months to report on local recurrence rate and disease-free survival. For conclusive results to be made, a larger cohort should be reviewed. This preliminary case-matched cohort study, however, shows promising short-term results. Hesitation to undertake TAMIS in cases of rectal polyps/carcinoma due to fear of completion surgery with worse pathological outcome and increased morbidity may be omitted by the introduction of completion TaTME. Instead of immediate TME when stage I carcinoma is expected, a low threshold for local therapy could be offered in early rectal cancer staged with MRI (T1/2 N0 with a diameter of < 3 cm) since completion surgery with TaTME seems feasible and safe and has favorable short-term outcomes. The majority will have low-risk features and for the minority indication for additional completion TaTME in this step-up approach should be considered [25]. For these patients with poor-risk features after TAMIS, a rectal preserving treatment option is currently under investigation in the TESAR trial, which is a randomized clinical trial comparing radical completion surgery versus adjuvant chemoradiation after TAMIS [26]. Until these results are available, completion surgery will be the golden standard for rectal cancer surgery and therefore, based on our results, transanal approach should be considered in these patients.

In conclusion, completion surgery with TaTME after a primary local excision for rectal cancers leads to significantly better quality of specimen, less perforation of the rectum, lower conversion rate, increased number of harvested lymph nodes, and shorter hospital stay compared to abdominal completion TME and has comparable pathological and short-term outcomes compared to primary TME. These results strengthen the step-up strategy of local excision followed by TaTME, if indicated based on pathology. Future studies are needed to evaluate potential differences in long-term functional and oncological outcomes between conventional TME and TaTME after full-thickness local excision.
